# The Effect of Cinnamaldehyde on iNOS Activity and NO-Induced Islet Insulin Secretion in High-Fat-Diet Rats

**DOI:** 10.1155/2021/9970678

**Published:** 2021-07-13

**Authors:** Zomorrod Ataie, Mohammad Dastjerdi, Khadijeh Farrokhfall, Zahra Ghiravani

**Affiliations:** ^1^Islamic Azad University, Zahedan Branch, Faculty of Medicine, Zahedan, Iran; ^2^Medical Toxicology and Drug Abuse Research Center (MTDRC), Birjand University of Medical Sciences, Birjand, Iran; ^3^Cardiovascular Diseases Research Center, Birjand University of Medical Sciences, Birjand, Iran

## Abstract

**Introduction:**

Obesity and insulin resistance are associated with alterations in nitric oxide level and insulin secretion. Previous studies demonstrated that cinnamaldehyde (CNMA) improved islet insulin secretion and restored nitric oxide (NO) level, but its underlying mechanisms have not been investigated. This study aimed to investigate the effect of CNMA on inducible nitric oxide synthase (iNOS) activity and NO-induced islet insulin secretion in high-fat-diet (HFD) treated rats.

**Materials and Methods:**

Forty male Wistar rats (12 weeks old) were randomly divided into four equal groups, namely, control, CNMA, HFD, and HFD + CNMA. Control and CNMA groups were treated with standard laboratory animals' diet, while HFD and HDF + CNMA groups were fed with an HFD diet enriched with 25% *W*/*W* tail fat for 16 weeks. CNMA was administrated orally (20 mg/kg body weight, daily) during the study period. Islet insulin secretion and the inducible NOS activity in the presence or absence of L-NAME (NO synthase inhibitor, 5 mmol/L) were evaluated.

**Results:**

L-NAME-suppressed insulin secretion in control, HFD, and HFD + CNMA groups; however, in the CNMA group, it could not exhibit such effect (*P* < 0.01). Islets of HFD-treated animals showed significantly higher iNOS activity than controls. CNMA treatment significantly suppressed iNOS activities in CNMA and HFD + CNMA groups compared with control and HFD, respectively.

**Conclusion:**

These results suggest that the beneficial effect of CNMA on insulin secretion might be due to its inhibitory effect on iNOS activity.

## 1. Introduction

Metabolic syndrome (MS) is defined as a collection of interrelated risk factors that can predict type 2 diabetes mellitus, stroke, cardiovascular disease, and other health threats. These risk factors include abdominal obesity, hyperglycemia, dyslipidemia, and high blood pressure with a core component of insulin resistance (IR). The pathophysiology of MS is not well understood. However, it is well known that nitric oxide (NO) plays a crucial role in the pathogeneses of metabolic syndrome [1] and is closely linked with insulin signaling [2]. Also, apparent data suggest that NO is involved in the development of IR and type 2 diabetes [3, 4]. On the other hand, NO has long been used as a vasodilator, and nitrate compounds are frequently used for angina, hypertension, and heart failure.

To study the pathophysiology of MS, numerous animal models of dietary approaches to imitate the disease condition in humans have been established. In this approach, single-type diets (high fructose, high sucrose, or high fat) or combinations of diets (high fructose/fat or high sucrose/fat) are frequently used [5]. Several high-fat diets (HFD) with different fat sources (plant- or animal-derived) as well as fat contents (vary between 20 and 60% of total energy) have been extensively used [5–7].

NO is a highly diffusible gaseous molecule with a short half-life in blood (a few seconds). NO plays essential roles such as cell signaling and vasodilator molecules in several biological processes. Therefore, its production is strictly controlled in almost every cell type [8]. The NO synthesis within cells is regulated by different enzyme isoforms and nonenzymatic pathways [9]. The enzymatic pathway is one of the most important ways in which NO production is regulated. In this pathway, NO formation is catalyzed by NO synthase (NOS) enzyme through a series of redox reactions in which NOS utilizes tetrahydrobiopterin, NADPH, and molecular oxygen to convert l-arginine to l-citrulline and NO. There are three isoforms of NOS in mammals: neuronal NOS (nNOS), endothelial NOS (eNOS), and inducible NOS (iNOS) that play important roles as dichotomous effects in human biology and diseases [8, 9].

In pathophysiological conditions such as diabetes, obesity, and insulin resistance, NOS activity is altered [10]. However, the NO production could be increased in hyperglycemia, but its bioavailability decreases due to degradation in reaction with free oxygen radicals [10]. Recent evidence demonstrated that alteration in iNOS expression is involved in the pathogenesis of IR, obesity, and diabetes [11, 12]. iNOS stimulation could produce a higher amount of NO than other isoforms of NOS (eNOS and nNOS) [11]. Despite the protective effects of NO in physiological concentration, its overproduction in pathophysiological conditions such as IR and obesity can lead to irreversible tissue damage [10–12]. Also, iNOS-derived NO primarily causes disturbance of carbohydrate metabolism through *β*-cell dysfunction and impaired insulin secretion [12]. NO also participates in insulin production and secretion. However, its role in insulin secretion is disputable [13]. It is revealed that high levels of NO inhibit the activity of cytochrome *c* oxidase and produce ATP, leading to impaired glucose-stimulated insulin secretion [12].

Owing to its short half-life, measuring nitric oxide is difficult. Therefore, assessments of plasma nitrate and nitrite (*NO*_*X*_) are being used as markers for the activities of NOS and the production of NO. There is a strong correlation between serum *NO*_*X*_ levels and NO production so that the determination of *NO*_*X*_ in the blood has been reported to be the most useful method of quantitation of NO production in the body [14].

Cinnamaldehyde (CNMA) can be isolated from several plant sources such as *Cinnamomum cassia* and *Cinnamomum burmannii.* The CNMA has been widely studied for its several pharmacological activities [15]. CNMA has many medicinal activities including antioxidant, antibacterial, anticancer, immunomodulatory, and antidiabetic activities [6, 16–18]. A part of the therapeutic effects of CNMA was accomplished by modulation in NO production [19]. As mentioned above, NO produced in islets of the pancreas participates in the synthesis and secretion of insulin. Moreover, there is evidence showing that CNMA could improve NO balance in cell lines [20] and islet insulin release in rats with metabolic syndrome [6]. However, the role of NO in the beneficial effects of CNMA on insulin secretion has not been studied directly. To illuminate this uncharted area, in the present study, we examined the NO pathway. Accordingly, the aim of this study was to investigate the impact of CNMA on iNOS activity and NO level in HFD-treated rats.

## 2. Materials and Methods

### 2.1. Animals and Sample Preparation

All procedures involving animals were performed according to the guides and rules in care and use of Laboratory Animals in Scientific Affairs with the Iranian Ministry of Health and Medical Education following the 1964 Helsinki declaration and its later amendments or comparable ethical standards. The animal experiments were approved by the Ethics Committee of Research Institute for Endocrine Sciences, Shahid Beheshti University of Medical Sciences (permit code: 91/08/168).

Male Wistar rats (12 weeks old, 220–250 g) were purchased from Pasteur Institute, Tehran, Iran. Animals were allowed to acclimatize for 14 days to the laboratory environment (12 h light/dark cycle, 30–35% humidity, and 20–24°C temperature) before the experiment. Afterwards, they were randomly divided into four groups of 10 rats each. Group 1 received standard diet (control); group 2 was treated with a semi-purified HFD (HFD); group 3 received standard diet plus 20 mg/kg (6) CNMA by daily gavage for 16 weeks (control + CNMA); and group 4 was treated with a semipurified HFD plus 20 mg/kg CNMA by daily gavage for 16 weeks (HFD + CNMA).

CNMA was purchased from Sigma Company (W228613, China) and was diluted with corn oil 2% (*v*/*v*). It was provided weekly and stored at room temperature and in a cold-dark place.

Semipurified HFD contained 25% (*w*/*w*) of fat (1% soya bean oil and 24% tail fat). The HFD was prepared monthly and conserved in a cold room at 4°C. Both regular and HFD were provided by Javaneh Khorasan animal food company (Mashhad, Iran). All animals had free access to their relative diets during the study period. At the final 16^th^ week of the study, overnight-fasted animals were anesthetized by sodium pentobarbital (60 mg/kg, intra-peritoneally) [21]. Blood samples were collected from the heart; plasma was obtained by centrifugation (5,000 rpm for 20 min at 4°C) for the evaluation of lipid profile, *NO*_*X*_, glucose, and insulin. Finally, the pancreas of animals was dissected out for islet isolation. The animals and their food weighed weekly. The food intake was calculated as food consumption (g/16 weeks) × diet energy (kcal/g).

### 2.2. Islet Isolation Protocol

The isolation of islets of Langerhans was performed as described previously [6]. Immediately after blood collection, the common bile duct was cannulated at the proximal end, and its opening to the duodenum was ligated, and then collagenase (Roche, Cat. Number 1213, Germany) solution (0.5 mg/ml in ice-cold Hanks' balanced salt solution, HBSS; pH 7.4) was injected into the common bile duct. Then, the pancreas was resected and incubated at 37°C for 10 min. Islets were hand-collected under a stereomicroscope (Kyowa Optical, SDZTR-PL model, Japan). Each incubation well contained five islets in 1.0 ml of Krebs–Ringer buffer (pH = 7.4) supplemented with 0.5 g/dl bovine serum albumin (BSA; Fluka, USA) and 16.7 mM glucose. They also were gassed with 95% O_2_ and 5% CO_2_ for 5 min to obtain constant pH and oxygenation. To investigate the role of NO, the incubation mediums of six wells of each group were supplied by different NOS inhibitors: aminoguanidine (AG) or L-NG-nitro arginine methyl ester (L-NAME, Sigma). All incubations were performed at 37°C for 60 min. Then, aliquots of the supernatant were removed and stored at −20°C for insulin measurement.

### 2.3. iNOS Activity Assay

To evaluate iNOS activity, 200 islets were collected and stored in 200 *μ*l lysis buffer containing ice-cold HEPES (20.0 mM; pH 7.4; Sigma), L-dithiothreitol (1.0 mM, Sigma), and protease inhibitor cocktail (1 tablet dissolved in 10 ml buffer; Cat. No. 11836153001, Roche Co., Germany) with EDTA supplement. The samples were stored at −80°C. On assay day, the islet aliquots were sonicated using 40% intensity for 10 seconds. After centrifugation at 35,000*g* for 10 min at 4°C, supernatants were used for protein and enzyme assays. Protein concentration was determined by the Bradford method. The islet samples were kept on ice at all times. For iNOS activity assay, 100 *μ*l of the supernatant was added to reaction solution composed of 20.0 mmol/L HEPES (pH 7.4), 0.2 mM L-arginine (Sigma), and 2 mM NADPH (Sigma) in a total volume of 0.5 ml and then incubated under constant air bubbling (1 ml/min) at 37°C for 120 min. The blank/control samples contained all reaction components except NADPH. Aliquots of the incubated islet samples (100 ml) were then passed through an Agilent 1100 series high-performance liquid chromatography (HPLC) system with a fluorescence detector for analysis of the L-citrulline formed. Because L-citrulline is produced in equimolar to NO concentrations and more stable than NO, it is considered as NO production level. iNOS activity was calculated from the different L-citrulline AUC (area under the curve) between samples and their respective blanks/controls. L-citrulline was applied as standard. The L-citrulline HPLC methods were precisely described in the previous publication [21].

### 2.4. Measurement of Plasma *NO*_*X*_ Concentration

For indirect measurement of NOS activity, nitric oxide metabolite (nitrite + nitrate) concentration was measured using the Griess protocol.

First, 50 *μ*L of zinc sulfate (15 mg/mL) was added to each sample (1 ml) and was shaken vigorously. After that, 100 *µ*L of vanadium III chloride (8 mg/mL) in 1 M HCl was added to each well. Eventually, 100 *µ*L of Griess reagent containing 50 *µ*L sulfanilamide (2%) and 50 *µ*L N-(1-Naphthyl)ethylenediamine dihydrochloride (0.1%) was added to each well. The plate was incubated for 20 min at 37°C, and absorbance was read at 540 nm. Sodium nitrate (0–150 *µ*M) was used as a standard [22]. The intra-assay coefficient of variation for plasma *NO*_*X*_ was 3.12%.

### 2.5. Insulin and the Biochemical Plasma Assay

Islet and plasma insulin were assayed using an ELISA kit (insulin; Mercodia, Sweden). Fasting plasma glucose (FPG) was measured by the glucose oxidase method (Zistchem Co., Iran). The triglyceride (TG), total cholesterol (TC), HDL, and LDL cholesterol were measured by commercially available kits (Zistchem Co., Iran). The intra-assay coefficients of variation for insulin, glucose, TG, and TC were 7.34, 1.12, 1.97, and 1.22, respectively.

### 2.6. Statistics

All analyses were validated by D'Agostino and Pearson (omnibus K2 test performed with Prism version 5) normality test. Bartlett's test was used for evaluating the homogeneity of variances between groups. Data on weight were analyzed by two-way repeated measure analysis of variance (RM-ANOVA). Parameters including HOMA-IR, adiposity index, plasma glucose, TG, TC, HDL, and LDL were analyzed by two-way ANOVA with the factors being HDF and CNMA. The Bonferroni test was used for post hoc comparison. Islet insulin secretion of each group was analyzed by one-way ANOVA. The statistical analysis was performed using the GraphPad Prism software (version 5). Results were expressed as means ± SD of triplicate experiments. A value of *P* < 0.05 was considered statistically significant. The sample size was precisely chosen based on previous HFD studies [6, 16, 21, 23, 24].

## 3. Results

### 3.1. Serum *NO*_*X*_ Levels

**Figure 1:**
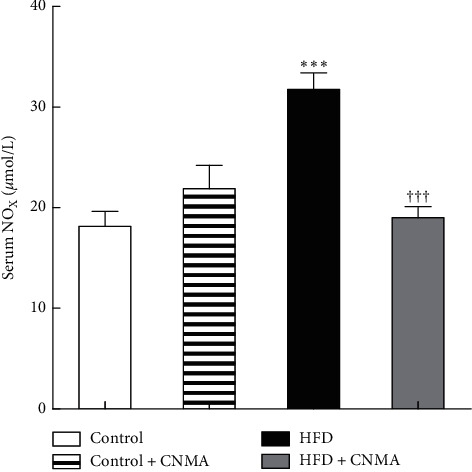
Serum *NO*_*X*_ (nitrite + nitrate) concentration in different experimental groups; *NO*_*X*_ was measured in serum of 10 rats in each group. Bartlett's test showed that variances are equal (*P* > 0.05) for all measures. Data were analyzed by one-way ANOVA and followed by the Bonferroni posttest. Results are presented as mean ± SEM. ^*∗∗∗*^Significant difference (*P* < 0.001) vs. controls and ^††^significant difference (*P* < 0.001) vs. HFD. All significant value was reported after calculation of Bonferroni corrections.

The results of plasma *NO*_*X*_ levels are presented in Figure 1. Plasma *NO*_*X*_ levels were varied according to diet (*P*=0.003) and CNMA treatment (*P*=0.01), with an interaction between diet VNMA (*P* < 0.001). *NO*_*X*_ level in the HFD group was significantly higher than control and other studied groups (*P* < 0.001). CNMA administration could effectively reduce (*P* < 0.01) as well as normalize plasma *NO*_*X*_ level in HFD-treated rats, which was similar to that of the control group. However, CNMA gavage in standard diet-treated animals did not exhibit a significant effect on plasma *NO*_*X*_ level.

### 3.2. Effects on Insulin Secretion

**Figure 2:**
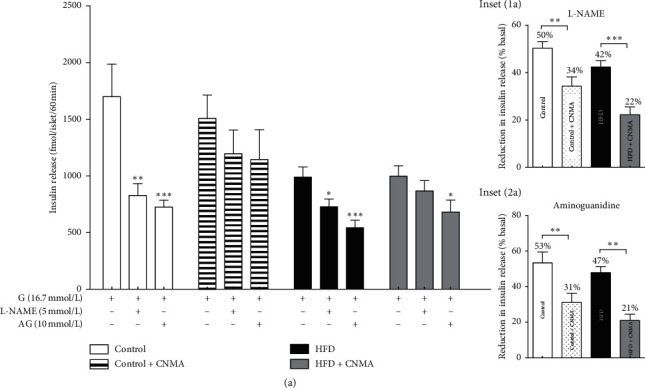
Effect of nitric oxide (NO) on insulin secretion of isolated islets from experimental groups. Insulin release was measured during 1 hour from groups of five islets at 16.8 mmol/L glucose alone and the presence of aminoguanidine (AG) and L-NAME after overnight fasting. Values are presented as mean ± SEM. for eight cups. Bartlett's test showed that variances are equal (*P* > 0.05) for all measures. Data were analyzed by one-way ANOVA and then the Bonferroni posttest. ^*∗*^*P* < 0.05, ^*∗∗*^*P* < 0.01, and ^*∗∗∗*^*P* < 0.001 compared with glucose 16.7 mmol/L in each group versus control in insets after the Bonferroni correction.

To investigate the effects of the two chemically different NOS inhibitors L-NAME and AG on islet insulin secretion, we assessed the insulin release from isolated islets incubated at 16.7 mM glucose in the presence/absence of these inhibitors. L-NAME supplementation (5 mmol/L) [2] significantly decreased insulin secretion from isolated islets belong to both HFD and control rats in the presence of 16.7 mmol/L glucose (Figure 2). Overall, at this high concentration of glucose (16.7 mmol/L), the insulin secretion was lower in islets of control (−50%) and HFD (−42%) groups. This effect was much more pronounced in the presence of L-NAME. The diet and CNMA consumption significantly reduced NO-induced islet insulin secretion (diet effect: *p*=0.01; CNMA effect: *P*=0.001). The addition of AG (10 mmol/L, 60 min incubation) [2] also markedly decreased insulin secretion from islets of both control and HFD groups (Figure 2). The results of two-way ANOVA revealed that CNMA significantly decreased islet insulin secretion in HFD and/or control groups (diet effect: *P*=0.105; CNMA effect: *P* < 0.0001). Similarly, the islet insulin release was lower at the presence of AG in the control (−53%) and HFD (−47%) groups (Figure 2). The results revealed that there was no difference in islet insulin secretion of the control + CNMA group in the presence or absence of either L-NAME or AG (Figure 2). Only AG supplementation significantly inhibited insulin secretion in the HFD + CNMA group.

The reduction ratio of insulin secretion was also calculated. Accordingly, the lowest reduction ratio of the islet insulin secretion was observed in CNMA-treated groups (Figure 2). In other words, these findings showing that CNMA consumption attenuated the role of NO in insulin secretion.

### 3.3. Results of iNOS Activity

**Figure 3:**
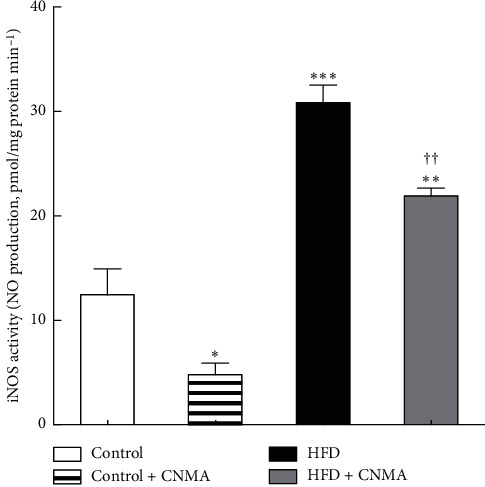
Comparison of iNOS (inducible nitric oxide synthase) activity in different experimental groups; iNOS activity was measured in six samples. The results are presented as mean ± SEM. Bartlett's test showed that variances are equal (*P* > 0.05) for all measures. Data were analyzed by one-way ANOVA and then the Bonferroni posttest. ^*∗*^, ^*∗∗*^, and ^*∗∗∗*^Significant differences (*P* < 0.05, *P* < 0.01, and *P* < 0.001, respectively) compared with the control group, and ^††^significant difference (*P* < 0.01) compared with the HFD group. The values remain significant even after Bonferroni corrections.

iNOS activity was determined as NO formation measured in the absence of Ca^2+^ and calmodulin (Figure 3). The iNOS activity varied according to diet (*P* < 0.001) and CNMA (*P* < 0.001); however, no statistically significant interaction was found between diet and CNMA on iNOS activity (*P*=0.52).

In comparison with control animals, HFD treatment during 16 weeks increased iNOS activity (*P* < 0.001). CNMA treatment markedly inhibited iNOS activity in islet of HFD-treated animals (*P* < 0.01) compared with the HFD-untreated group. However, the iNOS activity in HFD + CNMA group was still higher than the control group (*P* < 0.01; Figure 3).

## 4. Discussion

The present study aimed to investigate the effects of HFD and dietary CNMA supplementation on NO-induced islet insulin secretion and plasma *NO*_*X*_. The most important finding of this study was that CNMA supplementation reduced NO-induced islet insulin secretion and acted as an iNOS activity inhibitor irrespective of the diet. As shown in the present study, rats that consumed a high-fat diet developed metabolic syndrome criteria including insulin resistance, hyperglycemia, and obesity associated with increased body fat stores. Consistent with previous studies, our findings demonstrated that CNMA supplementation prevented insulin resistance and decreased plasma glucose level and adiposity in HFD rats [6, 16, 25, 26].

This study was performed to investigate the role of NO in antidiabetic activity of CNMA through assessments of plasma *NO*_*X*_ and islet iNOS activity as well as insulin secretion in normal and HFD-treated rats.

In all studied groups, insulin secretion decreased after NO synthesis inhibited with L-NAME; however, the reduction was only significant in untreated animals. NO has physiological importance in the biphasic secretion of insulin [27]. It is the main glucose regulator of islet insulin secretion [28]. The stimulatory effect of NO on insulin release is consistent with what has been found in previous reports [29–34], indicating that NO synthesis inhibition has diminished glucose-stimulated insulin secretion [32, 34]. Panagiotidis et al. have also reported that glucose-stimulated insulin secretion enhanced the intra-cellular concentration of Ca^++^, which in turn intrinsically increased the islet NOS activity. On the other hand, other studies have theorized that NO has an inhibitory effect on glucose-stimulated insulin secretion so that inhibition of NO synthesis stimulates insulin secretion [35, 36]. They have theorized many mechanisms, including the reduction in the activity of carbohydrates metabolism enzymes such as phosphofructokinase [36] and neutralization of the thiol/sulfhydryl groups of enzymes involved in insulin secretion. It has been emphasized that these effects are independent of cGMP [35, 37]. It has been demonstrated that NO increased the insulin gene expression in the short term (24 hours) and enhanced the *β*-cell function by PI 3-kinase signaling [38]. NO influences the activity of calcium channels and insulin release in a concentration-dependent manner. At low concentrations, NO facilitates insulin secretion through increasing the cGMP; however, at high concentrations, it has an inhibitory effect on insulin secretion [39, 40]. A previous electrophysiological study performed using NO donors on the beta cell line and primary beta cells has shown that NO also had a dual effect on the ATP-dependent potassium channel (K_ATP_) activity, that is, it increased K_ATP_ current and inhibited insulin secretion with its indirect effect. This effect was mediated by a decrease in the metabolism, the ATP/ADP ratio reduction, and then hyperpolarization of the beta cell membrane. However, NO, through directly affecting the K_ATP_, inhibited K_ATP_ current, depolarized the beta cell membrane, and consequently, increased insulin secretion [41]. NO has been interfered with the glycolysis [42] and citric acid cycle enzymes activity [43]. It has been revealed that NO is connected to the SH group (sulfhydryl group) of serum proteins and plasma membrane proteins [44]. It has also been proven that the factors connecting to the SH group directly inhibit the K_ATP_ currents of beta cells and insulin secretion [45]. Given the contradictory roles of NO on insulin secretion as well as its complicated mechanisms on the insulin vesicles release, we can conclude that in this study, NO had an insulin stimulation effect, and its importance remained strong following the HFD treatment.

We previously reported that the HFD decreased islet insulin secretion and CNMA significantly reduced insulin secretion and content in isolated islets of a high-fat diet [6]. The role of NO in insulin secretion seems to be the same in HFD and control islets. In the present study, the insulinotropic effect of NO in animals receiving CNMA was much less than control and HFD animals so that the rate of insulin secretion decreased in these islets after inhibition of NO. Accordingly, two hypotheses can be suggested: (1) the physiological role of CNMA on insulin secretion is similar to NO mimetic, and in consequence, the NO production due to enzyme activity was decreased and (2) CNMA acted as an NOS inhibitor. According to the results of the current study, the activity of the iNOS enzyme in animals consumed CNMA was lower than that of the control and HFD groups. Furthermore, plasma *NO*_*X*_ level in the HFD group confirms these results, and it seems to CNMA acted as an inhibitor of the iNOS enzyme. Other studies on the macrophage cell line (RAW 264/7) have shown that CNMA reduced the NO level and iNOS activity [20, 46, 47]. It also has been imposed that CNMA through anti-inflammatory effects (NF-*κ*B inhibition) could inhibit the NO production [47]. On the other hand, another study has investigated the vasorelaxant effect of CNMA on the mesenteric vascular bed and found that CNMA, similar to NO, could activate the NO/sGC/PKG signaling pathway as well as its downstream potassium channels [48].

In conclusion, HFD increased iNOS activity in islets. CNMA restored the stimulatory effect of NO on insulin secretion by normalized iNOS and serum *NO*_*X*_. Therefore, the part of beneficial effects of CNMA on carbohydrate metabolism was developed from the NO pathway by reducing islet iNOS activity.

This study has some limitations that should take into consideration for interpreting the data. First, we did not assess possible adverse effects of CNMA in other systems. Second, based on the experimental design, the reactive nitrogen species (RNS such as peroxynitrite (OONO−)) as well as its oxidative products including S-nitrosothiols and nitrotyrosine were not evaluated. Further experimental researches are required to reveal the exact NO pathway by which CNMA modulates islet insulin secretion.

## Data Availability

Data are available from the corresponding author upon request through the data access committee or institutional review board.

## Supplementary Materials

Supplementary Materials The composition of HFD was precisely described in Table 1 of supplementary data [6]. After 16 weeks of HFD ingestion, the metabolic syndrome criterion was clearly induced (obesity, hyperglycemia, and insulin resistance without any alteration in serum lipid profile). The data are reported in the supplementary file. Cinnamaldehyde gavage improved insulin resistance accompanied by restoration plasma insulin and glucose level as well as reduced weight gain due to HFD.
