# Supplementary material for: The Effect of Cinnamaldehyde on iNOS Activity and NO-Induced Islet Insulin Secretion in High-Fat-Diet Rats
Source: Evid Based Complement Alternat Med. 2021 Jul 13;2021:9970678. doi: 10.1155/2021/9970678 (PMC8292039; doi:10.1155/2021/9970678)
Supplement: Supplementary Materials — The composition of HFD was precisely described in Table 1 of supplementary data [6]. After 16 weeks of HFD ingestion, the metabolic syndrome criterion was clearly induced (obesity, hyperglycemia, and insulin resistance without any alteration in serum lipid profile). The data are reported in the supplementary file. Cinnamaldehyde gavage improved insulin resistance accompanied by restoration plasma insulin and glucose level as well as reduced weight gain due to HFD. [file 9970678.f1.doc]

**The metabolic syndrome induction by HFD**

The diet composition of normal and high tail fat diet has been shown in table S1. The complementary data that confirmed metabolic syndrome were presented in below. While initial mean of body weights was similar in all groups (244.4 ± 7.97), HFD treated animals exhibited a significant weight gain compared to controls (P<0.01, Fig S1). Total calorie intakes in HFD groups were significantly higher than those of the control group (Table S2, P< 0.001). The adiposity (body lipid index) was increased in the HFD compared to the other experimental groups. CNMA consumption reversed weight gain, calorie intake and caused a significant decline in adiposity in HFD treated rats (P< 0.05, table S2). Both diet and CNMA were significantly influenced insulin resistance index. The HOMA-IR of the HFD group was significantly higher than those of other experimental groups (P<0.001, Table S2). Based on these results, HFD induced IR and CNMA improved insulin sensitivity (P<0.01, Table S2). Moreover, HFD animals showed high levels of FPG and insulin. CNMA treatment could restore the alterations in the HFD treated animals (table S2).

**Table S1)** The composition of control and high fat diet

| **HFD** | **Control** | **Diet composition (g)** |
| --- | --- | --- |
| 458 | 658 | Carbohydrate |
| 180 | 200 | Protein |
| 10 | 10 | Soya bean oil |
| 240 | 0 | Tail fat |
| 80 | 100 | fiber |
| 32 | 32 | Microelements |
| 4.8 | 3.6 | Calorie/gram food |
| **1000** | **1000** | **Total** |

**Table S2)** Anthropometrical parameters, HOMA-IR, plasma insulin, glucose, and lipid profiles of rats in different experimental groups

|  | **Groups** | | | | |
| --- | --- | --- | --- | --- | --- |
| **P value**   1. **Diet effect** 2. **CNMA effect** 3. **Diet˟ CNMA** | **HFD+CNMA**  **Mean ± (SD)** | **HFD**  **Mean ± (SD)** | **Control+CNMA**  **Mean ± (SD)** | **Control**  **Mean ± (SD)** | **Variable** |
| 1. P=0.031 2. P<0.001 3. P=0.012 | 3.12 (0.72)‡ | 4.29 (1.11)*** | 2.84 (1.00) | 2.58 (0.57) | Adiposity index (% BW) |
| 1. P<0.001 2. P=0.11 3. P=0.19 | 7684 (618.8) | 8090 (563.6)*** | 6860 (260.3) | 6871 (457.6) | Energy intake (kcal/rat/16 weeks) |
| 1. P<0.001 2. P<0.001 3. P=0.006 | 5.78 (1.40) ‡‡‡‡‡‡ | 15.69 (6.60)*** | 3.84 (2.26) | 3.87 (1.32) | HOMA-IR |
| 1. P=0.002 2. P=0.002 3. P=0.009 | 111.20 (11.72)‡‡‡ | 160.30 (38.83)** | 107.80 (10.17) | 104.70 (18.00) | FPG (mg/dl) |
| 1. P=0.014 2. P=0.003 3. P=0.062 | 109.80 (44.93) ‡ | 218.70 (61.57)** | 92.97 (37.2) | 109.60 (40.30) | Plasma insulin (pmol/l) |
|  | **Lipid profile** | | | | |
| 1. P=0.142 2. P=0.702 3. P=0.950 | 80.58 (4.133) | 83.04 (8.666) | 73.55(6.636) | 76.69(7.482) | TC (mg/dl) |
| 1. P=0.209 2. P=0.063 3. P=0.877 | 119.8 (13.43) | 125.6 (13.95) | 118.0 (10.23) | 124.3 (12.85) | TG (mg/dl) |
| 1. P=0.098 2. P=0.061 3. P=0.384 | 43.76 (2.567) | 43.17 (2.549) | 43.00 (2.380) | 41.14 (1.457) | HDL (mg/dl) |
| 1. P=0.354 2. P=0.267 3. P=0.683 | 11.08 (5.215) | 12.24 (8.866) | 7.697 (6.411) | 10.66 (6.809) | LDL (mg/dl) |

FPG; fasting plasma glucose, HOMA-IR; homeostasis model assessment of insulin resistance, TC; total cholesterol, TG; triglyceride. HOMA-IR was calculated using fasting glucose and fasting insulin by following formula: HOMA-IR= [fasting insulin (μU/ml) ˟ fasting glucose (mM/l)]/22.5. The comparisons are significant after Bonferroni correction in 10 rats. *****Significant differences between HFD and controls groups. ^‡^ Significant differences between HFD and HFD+ Cinnamaldehyde (CNMA).

**Fig S1)** The effect of high fat diet (HFD) and cinnamaldehyde (CNMA) consumption on body weight. Values are mean± SEM. Data were analyzed by two-way RM- ANOVA. The significant value was presented due to Bonferroni correction.

*significant differences between HFD / HFD+CNMA and controls groups
